# Diagnostic and Therapeutic Challenges in Native Vertebral Osteomyelitis: A Case Report of Infection Following Spinal Platelet-Rich Plasma Injection

**DOI:** 10.7759/cureus.87775

**Published:** 2025-07-12

**Authors:** Sayf Adas, Arielle Friedman, Hussein Abourahma, Sagar J Patel, Cristina Savu

**Affiliations:** 1 Dr. Kiran C. Patel College of Osteopathic Medicine, Nova Southeastern University, Fort Lauderdale, USA; 2 Department of Internal Medicine, University of Pittsburgh Medical Center, Pittsburgh, USA; 3 Department of Internal Medicine, Broward Health Medical Center, Fort Lauderdale, USA

**Keywords:** chronic low back pain (clbp), empiric antibiotic therapy, iatrogenic infection, spinal prp injection, transaminitis, vancomycin flushing syndrome, vertebral discitis, vertebral osteomyelitis

## Abstract

Native vertebral osteomyelitis (NVO) is an infection of the vertebral bodies and intervertebral discs that can result in significant morbidity if diagnosis or treatment is delayed. A 35‑year‑old man with chronic low‑back pain underwent a lumbar platelet‑rich plasma (PRP) injection. Approximately four months after the procedure, he developed new-onset low‑back pain that progressively worsened over the course of four weeks, accompanied by intermittent fevers and bilateral leg weakness. Magnetic resonance imaging (MRI) revealed L4-L5 discitis with adjacent end‑plate involvement, consistent with NVO. Blood cultures were negative, and two computed tomography (CT) guided biopsies failed to yield sufficient material for pathogen identification. Empiric broad‑spectrum antimicrobial therapy was initiated. The patient's treatment course was complicated by drug‑induced transaminitis and vancomycin flushing syndrome, requiring several regimen modifications. He ultimately stabilized on intravenous daptomycin and levofloxacin and was discharged with plans for six weeks of outpatient therapy and close follow‑up. This case highlights PRP injection as a potential iatrogenic source of NVO. Clinicians should maintain a high index of suspicion for spinal infection in patients presenting with new or worsening back pain weeks to months after minimally invasive spinal procedures. Early imaging, multidisciplinary management, and patient counseling on post-procedure infection risks are critical, especially when culture data are unavailable and empiric therapy must be carefully adjusted over time.

## Introduction

Platelet-rich plasma (PRP) injections have become increasingly popular in the treatment of chronic low-back pain due to their regenerative potential and minimally invasive nature. While generally considered safe, emerging reports suggest that spinal procedures, including PRP injections, carry a small but clinically significant risk of infection, including native vertebral osteomyelitis (NVO).

NVO is an infection of the vertebral bodies and intervertebral discs that is most often caused by hematogenous spread but can also result from direct inoculation during spinal interventions. Iatrogenic sources, such as epidural injections, intradiscal therapies, and spinal surgeries, are increasingly recognized, particularly in patients without traditional risk factors like intravenous drug use, diabetes mellitus, or immunosuppression [1-2]. Although infection following PRP injection is rare, a 2022 clinical trial reported one case of discitis among 31 patients treated with intradiscal autologous PRP, underscoring the need for ongoing vigilance [3]. More recent systematic reviews have emphasized the importance of strict aseptic technique during PRP preparation and administration to reduce infection risk and have called for further research to define standardized protocols and long-term safety outcomes [4-6].

Diagnosing iatrogenic NVO can be challenging due to its often indolent presentation and the frequent absence of positive culture results. Magnetic resonance imaging (MRI) is the most sensitive imaging modality, particularly in the presence of elevated inflammatory markers such as erythrocyte sedimentation rate (ESR) and C-reactive protein (CRP). When blood cultures are negative, image-guided biopsy may be needed, although it often fails to yield a definitive pathogen [7].

This case illustrates PRP injection as a potential iatrogenic source of spinal infection and highlights the diagnostic complexity of culture-negative NVO in a young patient without predisposing risk factors. It emphasizes the importance of maintaining clinical suspicion in patients presenting with new or worsening back pain following minimally invasive spinal procedures, and the need for timely imaging and multidisciplinary care to guide management.

## Case presentation

Patient information

A 35-year-old Hispanic male patient with a medical history of gastroesophageal reflux disease (GERD) presented to the emergency department on November 11, 2024, with a four-week history of progressively worsening low back pain and recurrent fevers. These symptoms occurred four months after a PRP injection. The patient reported associated night sweats, bilateral leg weakness, and occasional electric shock-like sensations down both legs. There was no saddle anesthesia and no bowel or bladder incontinence. Over-the-counter flu remedies provided no symptom relief. Notably, the patient did not have health insurance at the time of presentation but stated it would start in January 2025. Written informed consent was obtained for all diagnostic procedures, including biopsy, and the off-label use of antibiotics described below.

Medical history

The patient’s medical history revealed no prior surgeries, hospitalizations, or allergies. At-home medications included ibuprofen, naproxen, acetaminophen, and topical diclofenac. Family history was unremarkable aside from a maternal grandmother with thyroid cancer. The patient’s social history revealed alcohol consumption (three to four drinks on weekends) and tobacco use (five cigarettes per week). 

Clinical course

**Figure 1:**
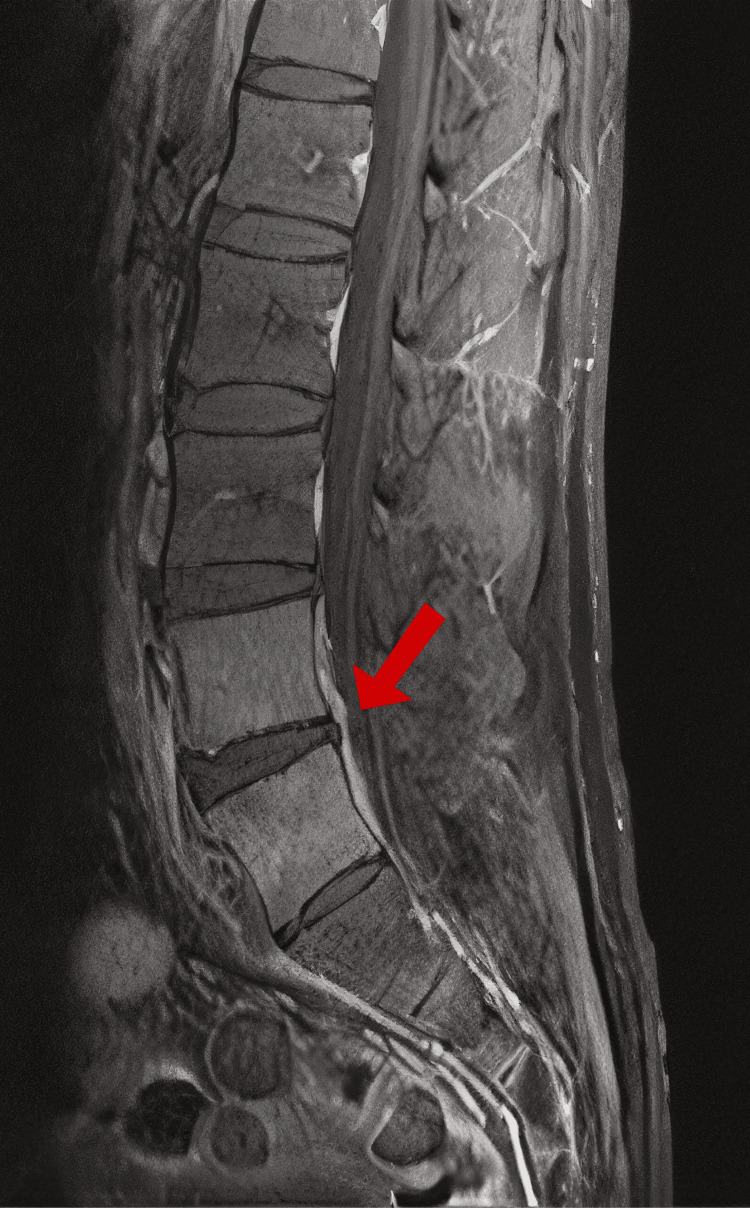
MRI lumbar spine, November 21, 2024 MRI: magnetic resonance imaging Sagittal T2-weighted MRI of the lumbar spine demonstrating hyperintense signal within the L4-L5 intervertebral disc and adjacent vertebral endplates with surrounding epidural soft-tissue thickening (arrow), findings consistent with vertebral discitis-osteomyelitis

The patient’s low-back pain began in the fall of 2023 following a work-related injury. MRI at that time showed a disc bulge without herniation, fracture, stenosis, or infection. He was prescribed a one-month course of oral steroids and a back brace. The pain persisted for six months, prompting a referral to an interventional pain management specialist, who administered a one-time PRP injection to the L4 and L5 vertebrae in August 2024. Notably, documentation regarding the sterile technique used during the PRP injection was incomplete, with no details provided on skin antisepsis, draping, or personal protective equipment. Throughout this time, the patient’s medical expenses were being paid for by their employer directly and not through an official workers' compensation program. By November 2024, the patient began experiencing progressive back pain, night sweats, bilateral leg weakness, and electric shock-like sensations in his legs. A subsequent MRI was obtained on November 21, 2024, which revealed vertebral discitis (Figure 1). Due to a lack of health insurance, the patient was advised to go to the emergency department the following day for further evaluation. 

Hospital course

Initial Presentation and Imaging

Upon admission, the patient was diaphoretic, with tenderness to palpation of the lumbar spine and hyperreflexia (3+ patellar reflexes bilaterally). A repeat MRI revealed osteomyelitis of the L4 and L5 vertebral bodies, with circumferential paravertebral and ventral epidural thickening, as well as a mild phlegmon. No abscess formation was identified.

Empiric Antimicrobial Therapy

Empiric intravenous (IV) vancomycin was initiated targeting Gram-positive organisms, including methicillin-resistant *Staphylococcus aureus* (MRSA). Based on infectious disease (ID) consultation and institutional protocols, IV meropenem and micafungin were added on hospital day two to provide broad coverage for possible Gram-negative bacterial and fungal pathogens.

Biopsy and Diagnostic Challenges

On hospital day five, a computed tomography (CT)-guided percutaneous biopsy was performed using a 17-gauge coaxial bone biopsy needle targeting the L4-L5 disc space. Unfortunately, the specimen was insufficient for culture and histopathologic analysis. Blood cultures drawn at 48 and 96 hours remained negative for growth. 

**Figure 2:**
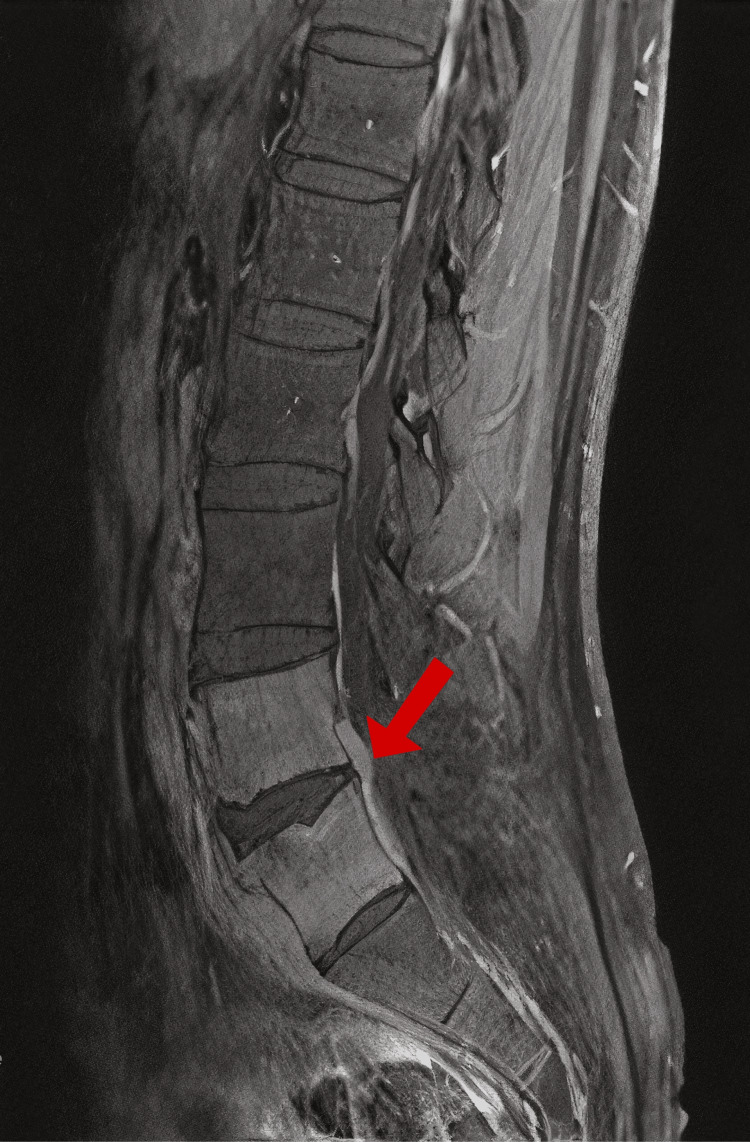
MRI lumbar spine, December 2, 2024 MRI: magnetic resonance imaging Sagittal T2-weighted MRI of the lumbar spine demonstrating persistent hyperintense signal in the L4-L5 intervertebral disc and adjacent vertebral endplates with surrounding epidural/paravertebral soft-tissue thickening (arrow)

**Figure 3:**
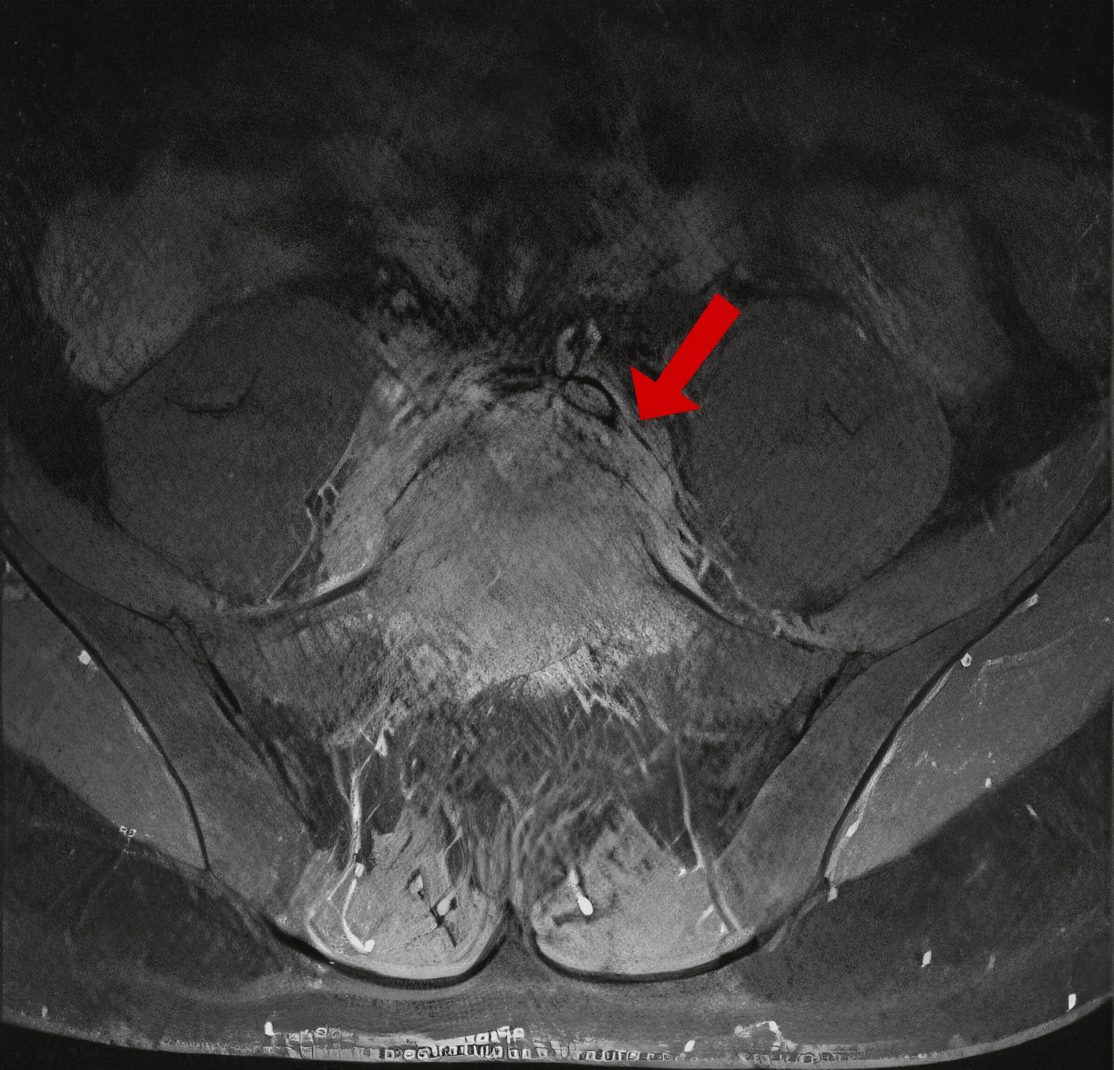
MRI lumbar spine, December 2, 2024 MRI: magnetic resonance imaging Axial T2-weighted MRI at the L4-L5 level showing circumferential epidural and paravertebral soft-tissue thickening (arrow)

A fourth MRI was obtained on hospital day 10 to assess whether there was progression of the phlegmon that might allow for a higher-yield open biopsy and culture. However, no significant changes were observed in the lumbar spine (Figures 2, 3). Given the absence of imaging progression and emerging concerns about the patient's overall condition, an open biopsy was deferred due to procedural risks. 

Treatment-Related Complications

By hospital day six, the patient developed transaminitis, with alanine aminotransferase (ALT) and aspartate aminotransferase (AST) rising to 88 units per liter (U/L) and 75 U/L, respectively. Due to worsening liver enzymes, meropenem was discontinued on day seven and replaced with ceftriaxone. Despite this, ALT and AST further increased to 214 U/L and 102 U/L by day 11, leading to ceftriaxone discontinuation. Concurrently, the patient developed a diffuse erythematous rash attributed to a vancomycin infusion reaction, consistent with vancomycin flushing syndrome. In response, all antibiotics were temporarily withheld.

Over the following 48 hours, transaminitis began to improve (ALT 174 U/L, AST 54 U/L), and the rash persisted without progression. On hospital day 14, IV antibiotics were resumed with daptomycin and levofloxacin as alternatives.

Monitoring and Discharge Planning

Inflammatory markers showed fluctuating trends, with ESR peaking at 105 millimeters per hour (mm/hr) and CRP declining from 5.13 to 2.51 milligrams per deciliter (mg/dL) during antibiotic-free intervals. Repeat MRI on hospital day 10 revealed no progression of the lumbar phlegmon. The patient remained neurologically stable. A summary of ESR, CRP, ALT, and AST values over the course of hospitalization is provided in Table 1. 

**Table 1:** Summary of inflammatory markers and liver enzymes during hospitalization ESR: erythrocyte sedimentation rate; CRP: C-reactive protein; ALT: alanine aminotransferase; AST: aspartate aminotransferase; mm/hr: millimeters per hour; mg/dL: milligrams per deciliter; U/L: units per liter; —: value not obtained

Hospital day	ESR (mm/hr)	CRP (mg/dL)	ALT (U/L)	AST (U/L)
3	55	5.13	—	—
6	105	3.89	88	75
10	—	—	170	95
11	85	2.51	214	102
13	—	—	174	54

**Figure 4:**
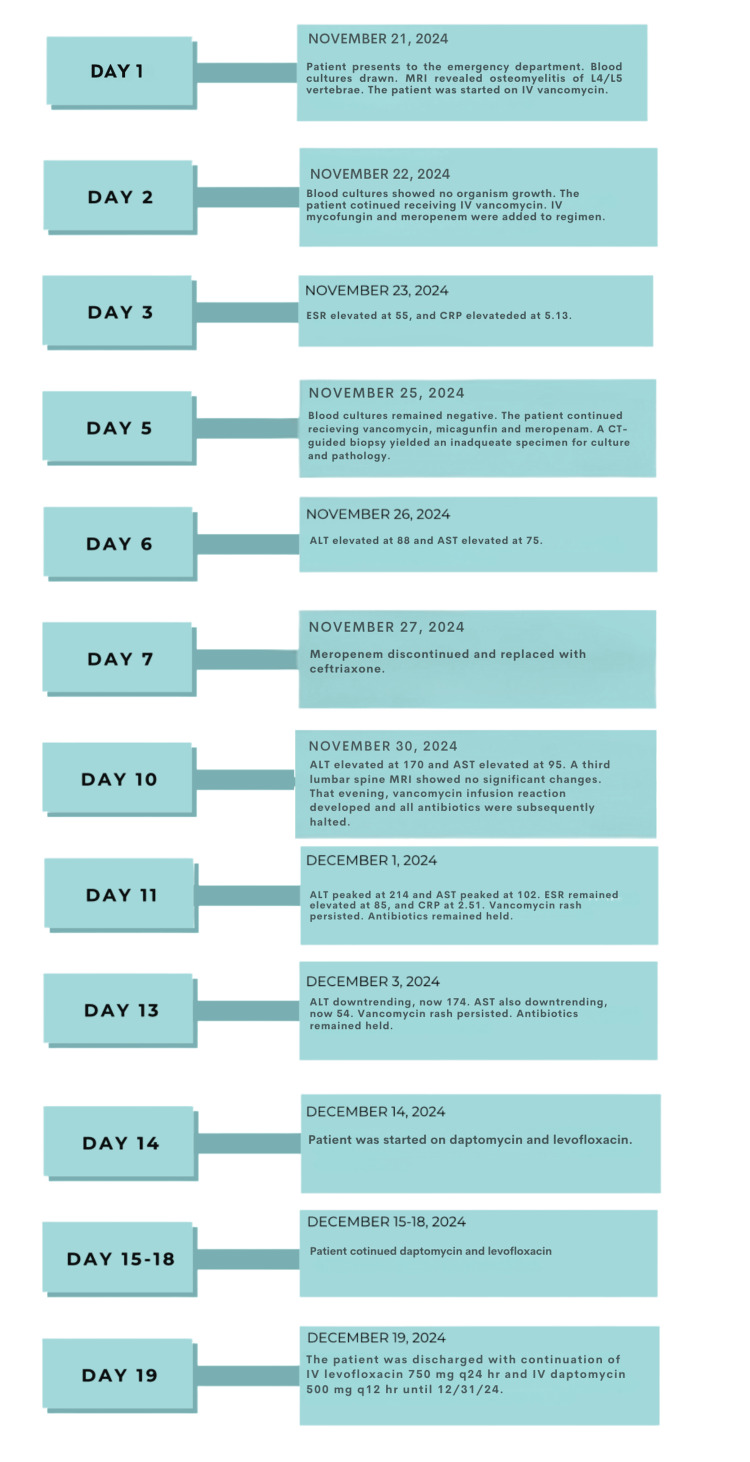
Timeline of hospital course and management MRI: magnetic resonance imaging; IV: intravenous; ESR: erythrocyte sedimentation rate; CRP: C-reactive protein; CT: computed tomography; ALT: alanine aminotransferase; AST: aspartate aminotransferase; mg: milligrams; q24 hr: every 24 hours; q12 hr: every 12 hours

After 19 days, the patient was discharged with a peripherally inserted central catheter (PICC) line for outpatient IV therapy with levofloxacin (750 mg every 24 hours) and daptomycin (500 mg every 12 hours) until December 31, 2024. Follow-up included weekly laboratory monitoring and a repeat MRI in four weeks. Figure 4 provides a visual summary of the hospital course, including key clinical events, diagnostic findings, and treatment changes throughout the 19-day admission.

## Discussion

Timely diagnosis and management of NVO can be complicated by an insidious onset of symptoms in the setting of pre-existing low back pain, as illustrated by a young patient with no prior risk factors who developed L4-L5 vertebral osteomyelitis four months after receiving a spinal PRP injection. Ultimately, the patient’s recent history of intermittent fevers and elevated inflammatory markers is what prompted diagnostic imaging. MRI remains the gold standard for detecting vertebral osteomyelitis, as plain radiographs are often normal in early stages [7]. Although imaging was obtained promptly once suspicion arose, the patient had already experienced weeks of worsening symptoms, highlighting the need to consider NVO in patients whose chronic back pain worsens disproportionately from baseline. 

While less common than hematogenous dissemination, the infection in this case was most likely due to direct inoculation following spinal injection. Although PRP-associated vertebral infections are rare, a small but growing number of case reports and reviews have documented this complication. A 2019 report described *Cutibacterium acnes* discitis after an intradiscal PRP procedure, marking one of the earliest microbiologically confirmed infections following orthobiologic therapy [8]. A 2020 case similarly reported culture-negative lumbar osteomyelitis after a stem cell-based spinal injection, which, like the present example, lacked definitive pathogen identification and required empiric treatment [9]. A 2021 review of intradiscal orthobiologic injections, including PRP and bone marrow concentrate, described two presumed and one confirmed infectious complication, underscoring the importance of rigorous aseptic technique [10]. More recently, a 2023 systematic review characterized PRP-related spinal infections as "very uncommon" but acknowledged that true incidence may be underreported, especially in settings without regulatory oversight or standardized procedural documentation [4]. This report contributes to the limited but important growing body of evidence, highlighting the potential for deep spinal infections even with minimally invasive, autologous biologic injections.

A key limitation of this case was the absence of microbiological confirmation, which necessitated empiric therapy. Without a confirmed pathogen, antimicrobial regimens cannot be tailored to organism-specific resistance profiles, increasing the risk of both undertreatment and drug-related toxicity. The inability to isolate an organism also limits a broader understanding of the microbial spectrum in post-PRP discitis. In this patient, reliance on broad-spectrum antibiotics led to complications such as transaminitis and infusion reactions. These adverse effects further complicated the hospital course and highlight the need for larger studies to inform empiric treatment protocols in culture-negative vertebral osteomyelitis.

Finally, the clinical trajectory was significantly influenced by issues of healthcare access. The patient's uninsured status delayed evaluation, limited initial treatment choices, and contributed to the decision to pursue PRP therapy rather than first-line physical therapy. This underscores the broader need for systemic reforms that improve insurance coverage, especially for patients with work-related injuries, in order to ensure timely and evidence-based care. Barriers to diagnostic imaging and procedural follow-up can delay diagnoses, lead to avoidable complications, and result in more costly interventions later in the disease course.

## Conclusions

This case highlights the challenges of diagnosing and managing NVO, particularly when it arises from less common routes of infection, such as direct inoculation from procedures like PRP injections. While hematogenous dissemination remains the primary cause of NVO, it is crucial to maintain a high index of suspicion for iatrogenic spread, as demonstrated here. Despite prophylactic antibiotic use, infection risk persists, underscoring the importance of strict sterile technique and patient education on potential complications. Conservative treatment with physical therapy should be prioritized before considering more invasive measures, especially in patients with chronic low back pain.

The patient’s uninsured status influenced their treatment decisions, reflecting the broader impact of healthcare accessibility on medical outcomes. Delayed diagnosis, empiric antimicrobial therapy due to the inability to isolate the pathogen, adverse drug reactions, and prolonged hospitalization were key challenges in this case. Clinicians should remain aware of both hematogenous and nonhematogenous sources of infection and consider early referral for back pain that does not respond to conservative interventions. Ultimately, this case underscores the importance of early recognition, targeted antimicrobial therapy, and comprehensive patient care to optimize outcomes, while highlighting the potential risks associated with spinal injections. The potential risks still require further investigation to better understand optimal management strategies and long-term outcomes. A structured course of care should be developed to better guide clinicians when encountering similar clinical scenarios. 
